# Nanopore Structure and Fractal Characteristics of Lacustrine Shale: Implications for Shale Gas Storage and Production Potential

**DOI:** 10.3390/nano9030390

**Published:** 2019-03-07

**Authors:** Lei Chen, Zhenxue Jiang, Shu Jiang, Keyu Liu, Wei Yang, Jingqiang Tan, Fenglin Gao

**Affiliations:** 1 State Key Laboratory of Petroleum Resources and Prospecting, China University of Petroleum, Beijing 102249, China; chenlei19880804@163.com (L.C.); u6016291@utah.edu (F.G.); 2 Unconventional Oil & Gas Cooperative Innovation Center, China University of Petroleum, Beijing 102249, China; 3 Energy & Geoscience Institute, University of Utah, Salt Lake City, UT 84108, USA; 4 Key Laboratory of Tectonics and Petroleum Resources, Ministry of Education, China University of Geosciences, Wuhan 430074, China; 5 School of Geosciences, China University of Petroleum, Qingdao 266580, Shandong, China; keyu_liu@icloud.com; 6 School of Geosciences and Info-physics, Central South University, Changsha 410012, China; tanjingqiang@aliyun.com

**Keywords:** shale gas, pore structure, fractal characteristics, lacustrine shale, Sichuan Basin

## Abstract

In order to better understand nanopore structure and fractal characteristics of lacustrine shale, nine shale samples from the Da’anzhai Member of Lower Jurassic Ziliujing Formation in the Sichuan Basin, southwestern (SW) China were investigated by total organic carbon (TOC) analysis, X-ray diffraction (XRD) analysis, field emission scanning electron microscopy (FE-SEM), and low-pressure N_2_ adsorption. Two fractal dimensions D_1_ and D_2_ (at the relative pressure of 0–0.5 and 0.5–1, respectively) were calculated from N_2_ adsorption isotherms using the Frenkel–Halsey–Hill (FHH) equation. The pore structure of the Lower Jurassic lacustrine shale was characterized, and the fractal characteristics and their controlling factors were investigated. Then the effect of fractal dimensions on shale gas storage and production potential was discussed. The results indicate that: (1) Pore types in shale are mainly organic-matter (OM) and interparticle (interP) pores, along with a small amount of intraparticle (intraP) pores, and that not all grains of OM have the same porosity. The Brunauer–Emmett–Teller (BET) surface areas of shale samples range from 4.10 to 8.38 m^2^/g, the density-functional-theory (DFT) pore volumes range from 0.0076 to 0.0128 cm^3^/g, and average pore diameters range from 5.56 to 10.48 nm. (2) The BET surface area shows a positive correlation with clay minerals content and quartz content, but no obvious relationship with TOC content. The DFT pore volume shows a positive correlation with TOC content and clay minerals content, but a negative relationship with quartz content. In addition, the average pore diameter shows a positive correlation with TOC content and a negative relationship with quartz content, but no obvious relationship with clay minerals content. (3) Fractal dimension D_1_ is mainly closely associated with the specific surface area of shale, suggesting that D_1_ may represent the pore surface fractal dimension. Whereas fractal dimension D_2_ is sensitive to multiple parameters including the specific surface area, pore volume, and average pore diameter, suggesting that D_2_ may represent the pore structure fractal dimension. (4) Shale with a large fractal dimension D_1_ and a moderate fractal dimension D_2_ has a strong capacity to store both adsorbed gas and free gas, and it also facilitates the exploitation and production of shale gas.

## 1. Introduction

With a severe energy shortage situation, increasing shale gas has received more attention worldwide, and a global astounding “shale gas green revolution” is booming in the past few years. More than 20 countries except for the North America are conducting prophase evaluation and pilot test of shale gas exploration and development, especially in China [1,2,3,4,5]. Shale gas resource has already become a natural gas production target with great potential and of great significance. In order to encourage and accelerate the exploration of shale gas, the Government of China introduced relevant policies to take shale gas as a new type of mineral resource in 2011.

The shale reservoir is an extremely tight reservoir characterized by strong heterogeneous, ultra-low porosity and permeability with abundant nanoscale pores [6,7,8]. Except that a small amount of shale gas occurs as dissolved gas in residual oil and water, most of shale gas occurs as adsorbed gas in organic matters and clay minerals or occurs as free gas in pores and fractures [9,10,11,12]. Shale reservoir research is one of the important tasks in the geological evaluation of shale gas [1]. It can provide a sufficient basis for shale gas exploration and development by investigating the reservoir spatial distribution characteristics and the occurrence state of natural gas. The pore structure characteristics are important factors in determining shale gas storage capacity and are one of the key parameters for shale reservoir evaluation and shale gas resources evaluation [9,11,13,14,15]. The pore structure of shale can be characterized by multiple parameters such as pore size, pore morphology, pore volume, specific surface area, and spatial distribution [6,7,11,15,16,17,18]. Accurately measuring these pore structure parameters and investigating their controlling factors are of great significance for the study of the shale gas accumulation mechanism.

At present, many methods can be used to investigate the pore structure characteristics of shale. Through scanning electron microscopy (SEM), field emission scanning electron microscopy (FE-SEM), environmental scanning electron microscopy (ESEM), focused ion beam-scanning electron microscopy (FIB-SEM), broad ion beam-scanning electron microscopy (BIB-SEM), transmission electron microscopy (TEM), nano-computed tomography (nano-CT), and other high-resolution observation equipment, the morphology, size, distribution, and connectivity of pores can be observed, and the qualitative characterization of pore structure of shale can be achieved [6,7,8,14,15,17,18,19,20,21,22,23,24,25,26]. Low-pressure gas adsorption, high-pressure mercury intrusion porosimetry (MIP), nuclear magnetic resonance (NMR), and small-angle neutron scattering (SANS) methods can not only quantitatively characterize the pore size distribution of shale, but also obtain the total pore volume, specific surface area, and other parameters [9,15,16,27,28,29,30,31,32,33,34]. Among them, low-pressure N_2_ adsorption has been proved highly effective in the pore structure characterization of shale. In recent years, more and more research have been conducted on the pore fractal characteristics of shale using N_2_ adsorption isotherms [35,36,37,38,39,40,41].

Fractal theory is a nonlinear mathematical theory firstly introduced by Mandelbrot in 1975 [42]. Now it has become a powerful tool to characterize the pore surface and pore structure characteristics of shale. In recent years, with the large-scale development of shale gas in North America and China, some scholars have conducted exploratory studies on pore structure and fractal characteristics of shale reservoirs [36,37,38,43,44,45]. The Frenkel–Halsey–Hill (FHH) fractal theory provides a new method for the quantitative study of pore structure in shale, which can be used to obtain the spatial distribution characteristic parameters of pore structure [44,45]. In general, current studies mainly focus on marine shale [36,37,39,41,43,44], whereas limited research has been done on the pore structure and fractal characteristics in lacustrine shale. The lacustrine shale reservoir is usually characterized with a small single layer thickness and a large cumulative thickness [46]. For example, for the studied lacustrine shale in this paper, the effective thickness ranges from 30 m to 70 m. It varies frequently in lithology and is commonly interbedded with the sandstone or limestone due to frequent changes in the lake level [47]. The maturity of organic matter is lower than that of marine shale, and the types of organic matter are mainly types II_2_ and III, which are relatively poor in hydrogen [3,46]. Different sedimentary environments also lead to the differences in the pore characteristics between lacustrine shale and marine shale. Therefore, it is very necessary to investigate the pore structure and fractal characteristics to fully understand the geological characteristics of lacustrine shale.

This study focuses on the lacustrine shale from the Lower Jurassic Da’anzhai Member in the Sichuan Basin, SW China. In order to investigate the pore structure and fractal characteristics of shale, FE-SEM and low-pressure N_2_ adsorption experiments combined with the FHH theory for shale samples were selected to be conducted. The main objectives of this study were to: (1) characterize the pore structure of the Lower Jurassic lacustrine shale; (2) investigate the fractal characteristics and their controlling factors; and (3) discuss the effect of fractal dimensions on shale gas storage and production potential. Our studies can provide a new research idea and method for the assessment and exploitation of natural gas in the lacustrine shale reservoirs.

## 2. Samples and Methods

### 2.1. Geological Setting and Samples

**Figure 1:**
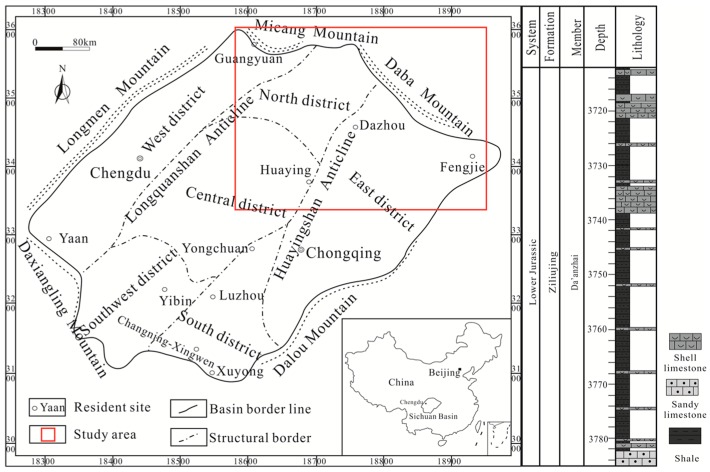
Study location, geological map, and simplified stratigraphic column of the Jurassic Da’anzhai Member in the northeastern Sichuan Basin.

The Sichuan Basin covers a vast area of the eastern Sichuan province and most of the Chongqing metropolitan region (Figure 1). It is a tectonically stable and superimposed petroliferous basin which is located in the western region of the Yangtze craton, SW China. It covers an area of more than 180,000 km^2^ with a diamond shape and a northeast trend. At present, a lot of oil fields and gas fields have been found in the Sichuan Basin. It is a multiple-cycle sedimentary basin which is bounded by the Longmen Mountain (fold belt) in the northwest, the Micang Mountain (uplift) and Daba Mountain (fold belt) in the north, and the Daxiangling and Dalou Mountains in the south [48].

The study area is located in the northeastern Sichuan Basin (Figure 1), which includes the Huaying and Dazhou areas. During the Jurassic Period, the Sichuan Basin was dominated by a lacustrine environment, and the Da’anzhai Member represents the largest and deepest stage of this environment [49], forming a large set of shale [47,49]. In addition, the Jurassic Da’anzhai Member shale formation varies frequently in lithology and is commonly interbedded with the shell limestone or sandy limestone due to frequent changes in the lake level during the Da’anzhai period (Figure 1). The studied shale samples originated from the Da’anzhai Member of Lower Jurassic Ziliujing Formation in the northeastern Sichuan Basin, SW China. All the shale samples were collected from fresh core materials. About 100–200 g were weighed for each sample, most of them were crushed to less than 60 mesh size with sufficiently mixed. Prior to the experiments, the samples were dried under vacuum at 110 °C for at least 12 h. Samples information such as ID, TOC and other geological parameters are listed in Table 1. 

### 2.2. X-ray Diffraction Analysis

The mineralogical composition of the shale samples with the usage of the Ultima IV full-automatic powder X-ray diffraction (XRD) analyzer (Rigaku, Tokyo, Japan) was completed at Experimental Research Center of East China Branch, SINOPEC (Nanjing, China). 

The shale sample to be tested was first crushed, then from which about 5 g was moved into a mortar before being ground to about 300 mesh. The ground sample was divided into two parts with one for XRD analysis and the other being left in reserve in case obvious human error occurred in the experimental test process. During the experiment, the qualitative analysis was performed to determine the sample composition using the standard powder diffraction data provided by the Joint Committee of Powder Diffraction Standards of the International Centre for Diffraction Data (JCPDS-ICDD). The quantitative analysis was carried out to obtain the mineral composition and content of the shale samples in which the K-value method of China National Standard (GB 5225-86) was adopted.

### 2.3. FE-SEM Observation

The FE-SEM observation was conducted in Institute of Geology and Geophysics, Chinese Academy of Sciences (Beijing, China). A NanoFab ORION microscope made by Carl Zeiss, Jena, Germany, was used to observe the pore morphology of shale samples. It could produce images of the pore structure with a high resolution of 0.8 nm (at 40 kV operating voltage and high-vacuum mode). Prior to the experiment, in order to produce a smooth sample surface, grinding and Ar-ion polishing were need to be conducted. This method avoids the damage of sample surface caused by mechanical polishing processes and preserves the real pore morphology of the sample surface.

### 2.4. Low-Pressure N_2_ Adsorption

Low-pressure (<0.127 MPa) N_2_ adsorption analyses were performed by using a Micromeritics^®^ Tristar II 3020 surface area analyzer (Micromeritics Instrument, Norcross, GA, USA). at State Key Laboratory of Heavy Oil Processing in China University of Petroleum, Beijing.

Shale sample aliquots weighing 1 to 2 g were analyzed with N_2_ to obtain information about microscopic pore structure. Samples were automatically degassed at about 110 °C under vacuum for about 14 h to remove adsorbed moisture and volatile matter before analyzing with N_2_. For quantifying N_2_ adsorption, the sample was kept at the temperature of liquid nitrogen (77.35 K at 101.3 kPa). The relative pressure (*P*/*P*_0_) for N_2_ adsorption ranges from 0.001 to 0.995. The instrument’s computer software automatically generates adsorption isotherms and calculates surface areas, pore volumes, and pore size distributions based on multiple adsorption theories [32,33,50]. 

## 3. Results

### 3.1. Mineralogical Composition Determination by XRD 

**Figure 2:**
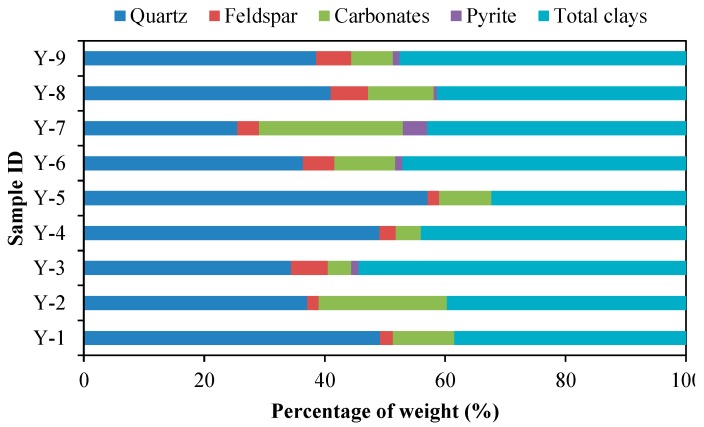
Mineralogical compositions of the shale samples.

As shown in Table 1 and Figure 2, the mineralogy of shale samples is complicated, and therefore the contents of each component from different samples vary significantly. The shale samples contain abundant quartz and clay minerals. The total clay minerals contents range from 32.3% to 54.4% with both temporal and spatial variation. The clay minerals generally reflect the diagenetic evolution and depositional environments and they are important to the study of methane storage capacity of shale. In addition to clay minerals, quartz is also the major mineral in the shale samples with a percentage of 25.5%–57.1%. Feldspar and carbonates are in significant quantities in the shale samples with a range of 1.9%–6.2% and 3.9%–23.9%, respectively. Pyrite occurs occasionally with a percentage of 0.6%–4.0%.

**Figure 3:**
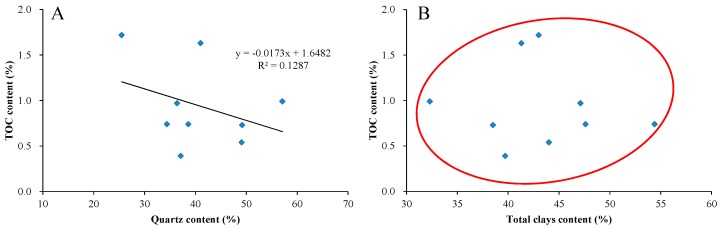
Plots showing the relationships of TOC content with quartz content (**A**) and total clays content (**B**) of the shale samples.

As shown in Figure 3A, TOC content shows a decreasing trend with the increase of quartz content, which is different from the strong positive correlation observed in marine shale samples [9,15,51,52,53]. This difference may be related to different quartz sources resulted from the differences in sedimentary environments of shale. The Da’anzhai Member shale is a product of lacustrine sedimentary environment and the quartz is primarily clastic in origin [49,54]. In addition, as shown in Figure 3B, there is no significant relationship between TOC content and total clays content, which may be related to the lacustrine sedimentary environment.

### 3.2. Pore Characteristics from FE-SEM Observation

**Figure 4:**
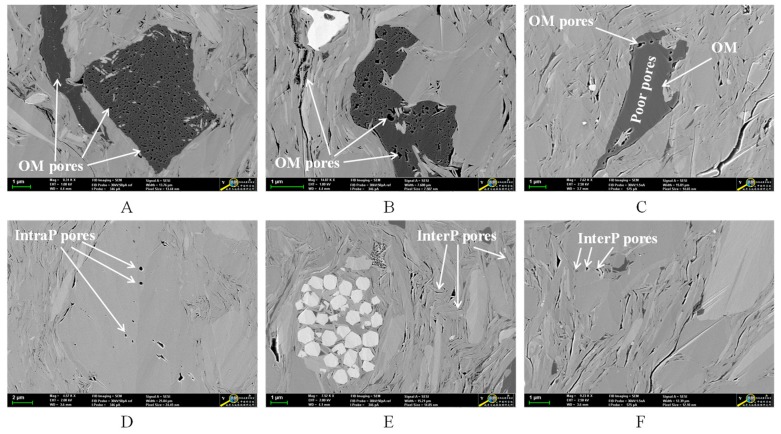
Field emission scanning electron microscopy (FE-SEM) images of micro-nanometer scale pores of the shale samples: (**A,B**) Abundant OM pores, (**C**) Heterogeneous OM pores characteristics in the same OM particle, (**D**) Abundant intraP pores, (**E**) Abundant interP pores between the clay minerals particles, (**F**) Abundant interP pores between the brittle minerals particles.

The FE-SEM images can be used to directly study the occurrence of pores in shale. Loucks et al. divided the pores into interparticle (interP) pores, intraparticle (intraP) pores, and organic-matter (OM) pores [7]. The interP and OM pores in the studied lacustrine shale samples are commonly developed (Figure 4). The OM pores are mainly controlled by the thermal evolution degree and type of organic matter [15,18,55]. With the increasing thermal evolution degree, the organic matter is transformed into hydrocarbon, thereby generating OM pores [6,18,21,56]. More specifically, the formation and preservation of OM pores are closely related to the evolution of kerogen. The evolution process of kerogen can be divided into the following three stages: (1) Diagenesis stage, in which kerogen is gradually formed with loose structure and low degree of polycondensation; (2) Catagenesis stage, in which kerogen begins to degrade, forming hydrocarbons, and its volume shrinks to form OM pores; and (3) Metagenesis stage, in which kerogen further polycondense to form carbon-rich residues, and OM pores gradually collapse or disappear. Such OM pores are mostly spherical or ellipsoidal, and some are honeycomb, mainly with tens to hundreds of nanometers, and usually have good connectivity (Figure 4A,B). In addition, a small number of OM pores are isolated, irregular in shape, and have a complex internal structure, mainly ranging from hundreds of nanometers to several micrometers. As shown in Figure 4C, the organic matter particles are large-sized, but only a few isolated OM pores are developed. Hu et al. and Yang et al. observed the same phenomenon when studying the Lower Silurian Longmaxi shale in the Sichuan Basin, China [36,44]. Curtis et al. pointed out that the reason for the different development of OM pores in organic matter particles which have undergone the same thermal evolution history is the difference in the type and composition of kerogen [18].

The main kerogen type in the studied shale samples is type III [49] and the thermal weightlessness in the thermal evolution process is lower than that in type I and type II kerogens, therefore, fewer OM pores are formed in the process of thermal evolution hydrocarbon generation. In addition, the maceral groups of type III kerogen are mainly vitrinite and inertinite, and the inertinite group has poor hydrocarbon generation potential, which also greatly inhibits the development of OM pores.

The interP and intraP pores are mainly developed in the mineral matrix (Figure 4D–F). Such pores are mainly controlled by diagenesis such as compaction, dissolution, and phase transformation of mineral [7]. Due to its strong plasticity, clay minerals are susceptible to compaction and bending deformation. Thus, the formed interP pores are mostly slit-shaped, polygonal, and strip-shaped (Figure 4E). The pores between the brittle minerals particles are mostly angular and mutually connected, which facilitates the migration and flow of shale gas (Figure 4F). The brittle minerals, clay minerals, and organic matters can form long and narrowly curved pores on the contact region of particles. There are also a certain amount of pores among pyrite crystals, which are mostly filled with organic matter and clay minerals. The FE-SEM observation shows that the development of OM pores in the study area is lower than that of Barrnet shale in North America [57] and Longmaxi shale in the southern Sichuan Basin, China [8]. Although the pores in clay minerals are relatively developed, they are affected by sedimentary facies change resulted from multi-period lake transgression and regression, as well as geological structure [47,49]. As a result, a part of the pores are distorted, damaged, and closed.

### 3.3. N_2_ Adsorption and Desorption Isotherms

**Figure 5:**
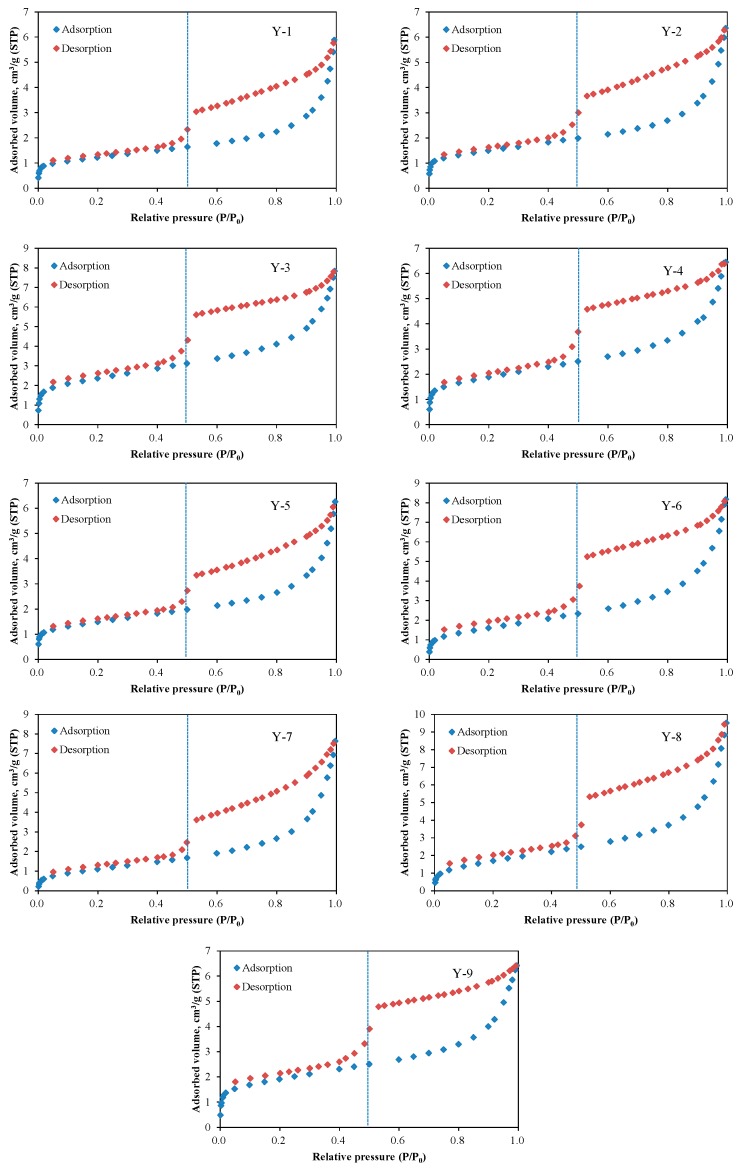
N_2_ adsorption and desorption isotherms of the shale samples: Y-1–Y-9 represent the sample ID.

Figure 5 shows the adsorption and desorption isotherms of N_2_ in shale samples. As illustrated in Figure 5, there is obvious hysteresis when relative pressure is higher than 0.5 (*P*/*P*_0_ > 0.5). Previous studies have already shown that the adsorption hysteresis occurs mainly due to capillary condensation in the mesopores and macropores [15,44,58,59]. According to the Kelvin equation, capillary condensation does not happen when relative pressure is lower than 0.5 (*P*/*P*_0_ < 0.5), in which range the adsorption and desorption curves overlap on each other [60]. As shown in Figure 5, the shapes of hysteresis loops vary with different shale samples, due to the different pore size distribution and pore geometries associated with shale samples. The discrepancies among these adsorption isotherms under different relative pressures reveal that the adsorption mechanisms are not the same. At lower relative pressures, gas adsorption mainly occurs in the micropores (less than 2 nm), where the interaction between gas molecules and shale pore surface molecules is strong, and dominated by Van der Waals force [61]. At higher relative pressures, more gas molecules have access to the mesopores (between 2 and 50 nm) and macropores (greater than 50 nm), and the interaction among gas molecules acts as the dominant force. As a result, gas adsorption predominantly relies on capillary condensation action [61]. Therefore, it is necessary to investigate the pore surface and structure characteristics of shale to understand their effects on gas adsorption in different pressure regions.

### 3.4. Pore Structure from N_2_ Adsorption Isotherms

**Figure 6:**
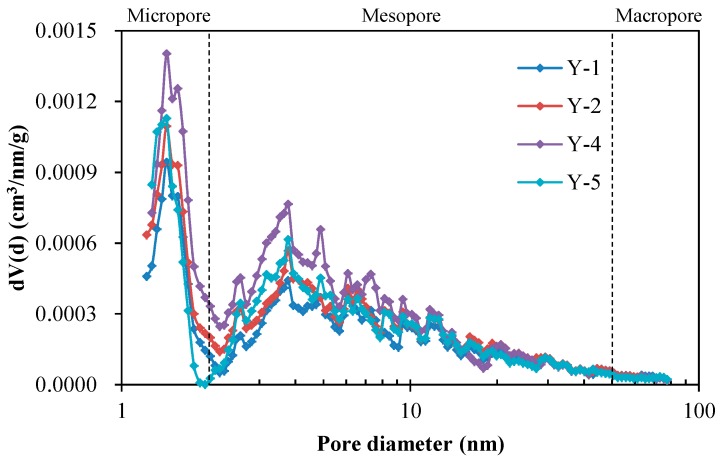
Pore size distributions (PSDs) obtained from N_2_ adsorption isotherms of four shale samples (Samples Y-1, Y-2, Y-4, and Y-5).

Pore structure parameters obtained from N_2_ adsorption isotherms are shown in Table 1. The Brunauer–Emmett–Teller (BET) surface areas of shale samples range from 4.10 to 8.38 m^2^/g with an average of 5.87 m^2^/g, much larger than the surface area of conventional sandstone, which is approximately 1 m^2^/g [62]. The specific surface area of shale is larger than sandstone because shale contains more clay minerals and organic matter, while clay minerals and organic matter have larger specific surface area than other minerals. The large specific surface area can provide sufficient adsorption sites for methane, which is beneficial to adsorbed gas storage. Previous studies show that the amount of adsorbed gas in shale accounts for 20% to 85% of the total gas content, with an average of about 50% [63]. According to the density-functional-theory (DFT) model, the pore size distributions (PSDs) of shale samples are shown in Figure 6. It can be seen that the pore size distribution curve of shale is complex and there are multiple different peaks, which may be related to the lacustrine sedimentary environment. The average pore diameters of shale samples range from 5.56 to 10.48 nm with an average of 7.39 nm. According to the International Union of Pure and Applied Chemistry (IUPAC) classification, the average pore diameter of shale falls into the range of mesopore. Furthermore, the shale contains a certain amount of macropores, resulting in a “tailing” phenomenon in the pore size distribution curve. The DFT pore volumes of shale samples range from 0.0076 to 0.0128 cm^3^/g with an average of 0.0098 cm^3^/g. Compared with the coal seam pores dominated by adsorbed gas, the pore volume of shale is one order of magnitude higher than that of coal seam, and larger pores are favorable for natural gas flow [61].

### 3.5. Fractal Dimensions from N_2_ Adsorption Isotherms

Fractal concept was first proposed in 1975, by Mandelbrot [42], and widely used in porous media including shale [35,36,37,38,39,40,41,43,44,45]. Fractal theory is used to describe the roughness of pore surface and the complexity of pore structure for porous media. It has been developed to be a powerful tool to analyze the geometric and structural characteristics of shale surfaces and pores. The commonly used parameter is fractal dimension D. The larger the fractal dimension, the rougher the pore surface or the more complex the pore structure. It is a well-accepted method to use gas adsorption for fractal dimension calculation [35,36,37,38,39,40,41,64]. According to Pfeifer’s theory [65], the fractal dimension can be calculated by the FHH equation:

(1) $$\ln V=K\ln\left[\ln\left(\frac{P_{0}}{P}\right)\right]+C$$

Where *V* represents the adsorbed volume (cm^3^) at equilibrium pressure *P* (MPa); *P*_0_ (MPa) is the vapor saturation pressure; *K* is the constant related to adsorption mechanism and fractal dimension *D*; *C* is a constant.

The relationship between *K* and *D* is:

(2) K=D−3.

**Figure 7:**
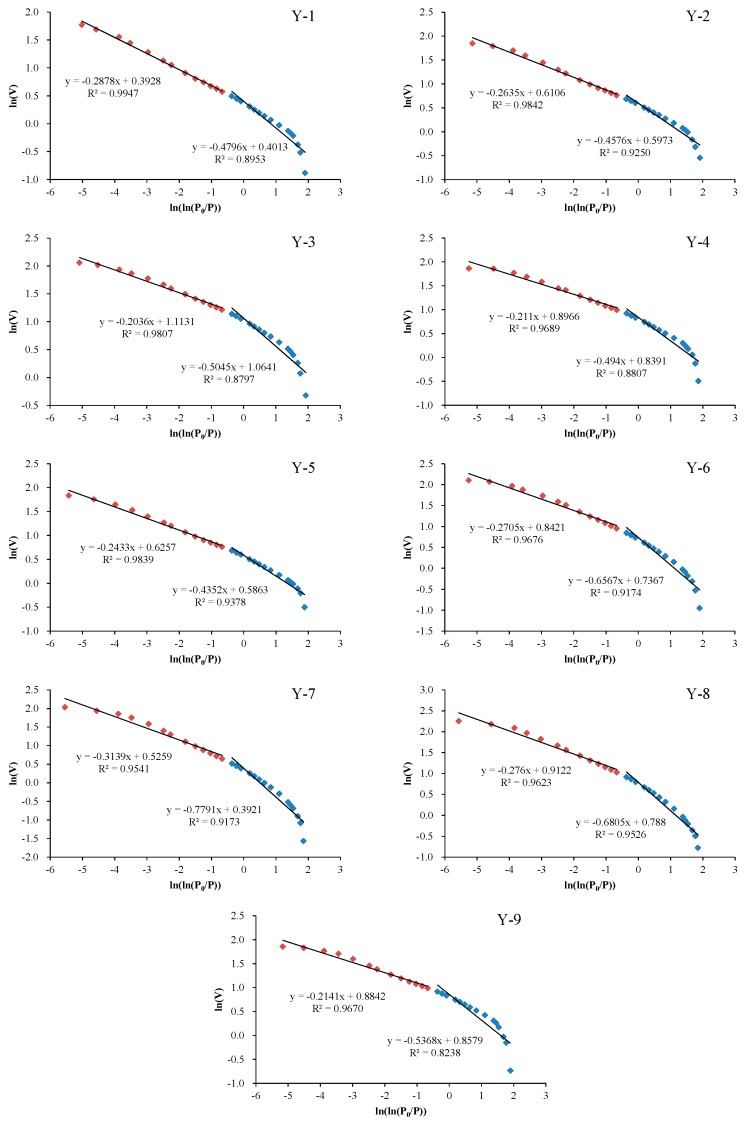
Plots of ln(V) vs ln(ln(*P*_0_/*P*)) reconstructed from N_2_ adsorption isotherms of the shale samples: Y-1–Y-9 represent the sample ID.

As discussed previously, N_2_ adsorption isotherm overlaps with desorption isotherm when relative pressure is lower than 0.5 (*P*/*P*_0_ < 0.5), the adsorption hysteresis occurs when relative pressure is higher than 0.5 (*P*/*P*_0_ > 0.5), indicating that the adsorption mechanisms before and after this relative pressure (*P*/*P*_0_ = 0.5) are different. Therefore, N_2_ adsorption and desorption isotherms in different pressure regions reflect different characteristics of shale. When calculating the fractal dimension with N_2_ adsorption data, it is necessary to calculate two fractal dimensions at relative pressures of 0–0.5 and 0.5–1, respectively, to characterize different characteristics of shale. Therefore, the fractal dimension calculation should be carried out separately at different pressures. In calculating the fractal dimension, regression of adsorption isotherms at low- and high-pressure segments were carried out separately, and two fractal dimension parameters D_1_ and D_2_ were generated accordingly. The results of linear regressions are shown in Figure 7 and Table 2. D_1_ values range from 2.2209 to 2.5648, which are generally lower than D_2_ values that are from 2.6861 to 2.7964. Previous studies illustrate that D_1_ and D_2_ represent two different fractal dimensions of shale [37,38,39]. D_1_ mainly represents the pore surface fractal dimension and is used to characterize the roughness of pore surface, however, D_2_ mainly represents the pore structure fractal dimension and is used to characterize the complexity of pore structure. 

## 4. Discussion

### 4.1. Specific Surface Area and Pore Volume Characteristics

**Figure 8:**
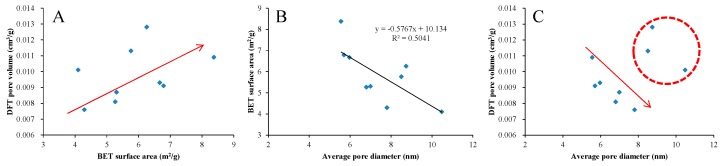
Relationships between density-functional-theory (DFT) pore volume and Brunauer–Emmett–Teller (BET) surface area (**A**), BET surface area and average pore diameter (**B**), DFT pore volume and average pore diameter (**C**).

**Figure 9:**
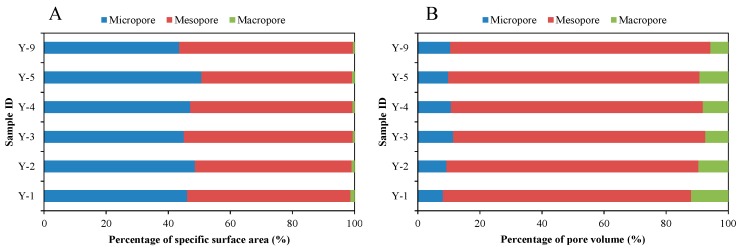
Percentages of specific surface area (**A**) and pore volume (**B**) of six typical shale samples.

Figure 8A shows that there is a significant positive correlation between DFT pore volume and BET surface area of the shale samples in the study area, and both the BET surface area and DFT pore volume have slight negative correlations with average pore diameter (Figure 8B,C). It should be noted that the decreasing trend of pore volume with the increase of average pore diameter is mainly reflected in the range of average pore diameter less than 8 nm, which may be related to the number distribution of pores with different diameters in the shale samples. Such correlations are similar to the study of lacustrine shale in the Yanchang Formation from the Ordos Basin reported by Jiang et al. [38] and Liu et al. [40]. They pointed out that such correlations indicate that the pore systems of lacustrine shale are mainly dominated by mesopores. As shown in Figure 9, the mesopores are also the main pores of the shale in the study area, providing 52.4% of total specific surface area and 81.4% of total pore volume. The micropores contribute 46.8% of total specific surface area and 9.9% of total pore volume, while the macropores contribute 0.8% and 8.7%, respectively. However, the correlations in marine shale are often different from that in lacustrine shale [66,67], which is mainly related to pore types and pore structures in marine and lacustrine shales [68]. The pores in marine shale are mainly derived from a large number of OM pores, while the OM pores mainly falls into the range of micropores. Therefore, they have relatively high specific surface areas and low pore volumes, resulting in the high content of adsorbed gas but low content of free gas in shale. Lacustrine shale not only contains a certain amount of OM nanopores but also a large amount of mineral-associated pores with relatively large pore diameters (Figure 4D–F). The mesopores can not only provide a certain pore volume, but also provide a considerable specific surface area. Therefore, the shale dominated by mesopores has a relatively strong storage capacity for free and adsorbed gas. 

### 4.2. Effects of TOC Content and Mineralogical Composition on Pore Structure 

**Figure 10:**
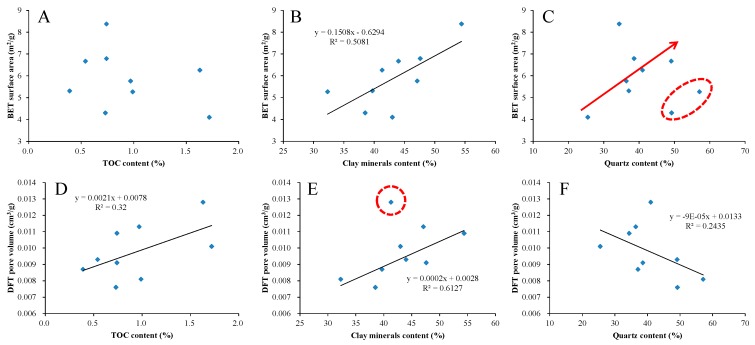
Plots of pore structure parameters (BET surface area and DFT pore volume) versus TOC content (**A**,**D**), clay minerals content (**B**,**E**), and quartz content (**C**,**F**).

In order to investigate the controlling factors of pore development from the Lower Jurassic lacustrine shale in the study area, the relationships between pore structure parameters (BET surface area and DFT pore volume) and TOC content and mineralogical composition are plotted in Figure 10. According to the results of XRD analysis, clay minerals and quartz are the most important mineral components of the shale in the study area. Therefore, clay minerals and quartz are mainly discussed in this study. It can be seen that the BET surface area shows no obvious correlation with TOC content, but it is positively correlated with clay minerals content and quartz content (Figure 10A–C). It should be noted that the increasing trend of specific surface area with the increase of quartz content is mainly reflected in the range of quartz content less than 50%, which may be related to the micropores number distribution in quartz of the shale samples. The DFT pore volume is positively correlated with TOC content and clay minerals content, but shows a weak negative correlation with quartz content (Figure 10D–F).

With the increase of TOC content, the OM pores increase, and the contribution to pore volume of shale also increases, but it has less effect on specific surface area. This may be due to the fact that the pores developed in organic matter of the Lower Jurassic lacustrine shale in the study area are mainly mesopores and macropores, which can also be confirmed by the FE-SEM images (Figure 4A,B). With the increase of clay minerals content, both the specific surface area and pore volume of shale increase, which may be due to the fact that clay minerals not only provide micropores, but also provide mesopores and macropores, which can be confirmed by previous studies [69]. As the quartz content increases, the specific surface area of shale increases, but the pore volume decreases. This may be due to the development of a large number of micropores in quartz, whereas the micropores have relatively large specific surface areas but relatively small pore volumes.

**Figure 11:**
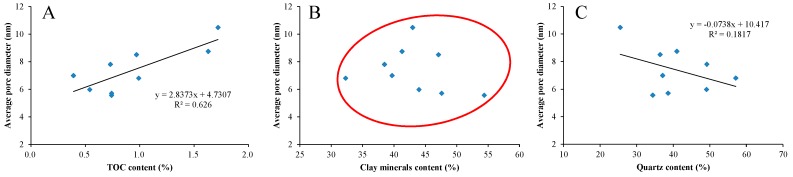
Plots of average pore diameter versus TOC content (**A**), clay minerals content (**B**) and quartz content (**C**).

The relationships between average pore diameter and TOC content and mineralogical composition are plotted in Figure 11. It can be seen that the average pore diameter has a positive correlation with TOC content (Figure 11A) and a weak negative correlation with quartz content (Figure 11C), but no obvious relationship with clay minerals content (Figure 11B). With the increase of TOC content, large pores (mesopores and macropores) increase in shale, resulting in the increase of average pore diameter. With the increase of quartz content, the increase of micropores in shale leads to a decrease in the average pore diameter, which is also observed in the Lower Silurian Longmaxi shale from the Sichuan Basin, China [43]. However, due to the simultaneous development of small and large pores in clay minerals, the effect on the average pore diameter is not obvious.

### 4.3. Relationships between Fractal Dimensions and TOC Content and Mineralogical Composition

**Figure 12:**
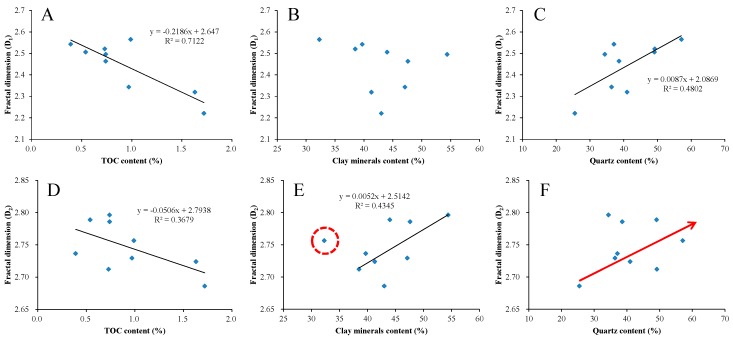
Plots of fractal dimensions D_1_ and D_2_ versus TOC content (**A**,**D**), clay minerals content (**B**,**E**), and quartz content (**C**,**F**).

Figure 12 shows the relationships between fractal dimensions (D_1_ and D_2_) and TOC content and mineralogical composition. The fractal dimension D_1_ shows a significant negative correlation with TOC content and a significant positive correlation with quartz content, but no obvious correlation with clay minerals content (Figure 12A–C). The fractal dimension D_2_ shows a weak negative correlation with TOC content, and a significant positive correlation with clay minerals content and quartz content (Figure 12D–F).

As previously mentioned, with the increase of TOC content, the number of large pores (mesopores and macropores) in shale increases, resulting in a decrease of pore surface and structure complexity in shale. As a result, both the fractal dimensions D_1_ and D_2_ decrease. This is inconsistent with the fact that the fractal dimensions of marine shale increase with the increasing TOC content [37,43]. It is mainly because of the high TOC content in marine shale and the development of numerous micropores in organic matter [15,28,70,71], leading to the increase of fractal dimensions of marine shale [37]. With the increase of clay minerals content, the number of micropores in shale increases, leading to a more complex pore structure and a larger fractal dimension D_2_. Meanwhile, the number of large pores in shale also increases, it can reduce the complexity of pore surface caused by the increase of micropores, resulting in no regular change in fractal dimension D_1_. With the increase of quartz content, the micropores in shale increase. As a result, the shale pore surface and structure become more complex, thus both the fractal dimensions D_1_ and D_2_ increase.

Therefore, it is suggested that clay minerals and quartz are the main controlling factors on the fractal dimensions of the Lower Jurassic lacustrine shale in the study area. In the study of fractal characteristics of marine shale, TOC content and quartz content usually act as the main controlling factors [37,43]. This is mainly due to the low content of clay minerals which has little effect on fractal dimension [41]. However, previous studies suggest that clay minerals have an important influence on the pore development of lacustrine shale [40,72]. The higher the content of clay minerals, the more developed the pores and the more complex the pore structure. It can be concluded that due to the differences of depositional environments, and the differences of mineralogical composition and organic matter characteristics, the pore development of shale is controlled by different factors. 

### 4.4. Relationships between Fractal Dimensions and Pore Structure Parameters

**Figure 13:**
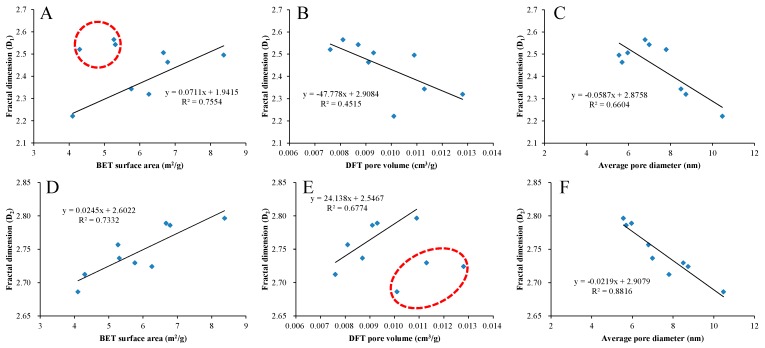
Plots of fractal dimensions D_1_ and D_2_ versus BET surface area (**A**,**D**), DFT pore volume (**B**,**E**), and average pore diameter (**C**,**F**).

**Figure 14:**
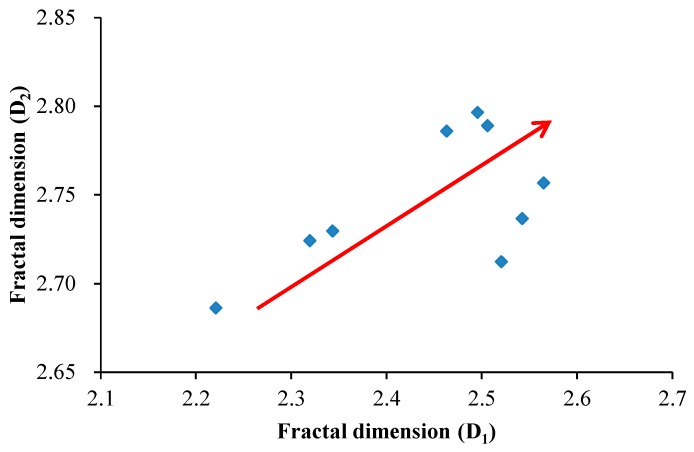
Plot showing the relationship between fractal dimension D_1_ and D_2._

The relationships between fractal dimensions and pore structure parameters (BET surface area, DFT pore volume and average pore diameter) of shale in the study area are illustrated in Figure 13. The fractal dimension D_1_ has a positive correlation with BET surface area and a negative correlation with DFT pore volume (Figure 13A,B). It should be noted that the increasing trend of fractal dimension D_1_ with the increase of BET surface area is mainly reflected in the range of BET surface area greater than 6 m^2^/g, which may be related to the tortuosity of pore surface in the shale samples. The fractal dimension D_2_ has a positive correlation with BET surface area and DFT pore volume (Figure 13D,E). It should also be noted that the increasing trend of fractal dimension D_2_ with the increase of DFT pore volume is mainly reflected in the range of DFT pore volume less than 0.011 cm^3^/g, which may be related to the distribution of pore throat in the shale samples. In addition, as shown in Figure 14, there is a good positive correlation between fractal dimension D_1_ and D_2_, which further indicates that D_1_ and D_2_ can jointly characterize the roughness of pore surface and the complexity of pore structure in shale, and they are not mutually independent. The increase of specific surface area indicates that the number of micropores increases, and the complexity of pore system increases, thus both the fractal dimensions D_1_ and D_2_ increase. The increase of pore volume indicates that the number of large pores increases, which reduces the complexity of pore surface and enhances the complexity of pore structure, thus the fractal dimension D_1_ decreases and D_2_ increases.

Both the fractal dimensions D_1_ and D_2_ decrease with the increase of average pore diameter (Figure 13C,F), but the average pore diameter exerts a greater influence on D_2_ than D_1_, suggesting that D_2_ may be more related to the pore structure fractal dimension, so it is more sensitive to the average pore diameter than D_1_. Shale samples with smaller average pore diameters may contain more micropores and throats [37,41,61], leading to more complex pore structures and higher fractal dimension D_2_. These micropores and throats increase the complexity of pore structure, which hinder the diffusion and flow of natural gas in the pore network [37].

### 4.5. Shale Gas Storage and Production Potential

**Figure 15:**
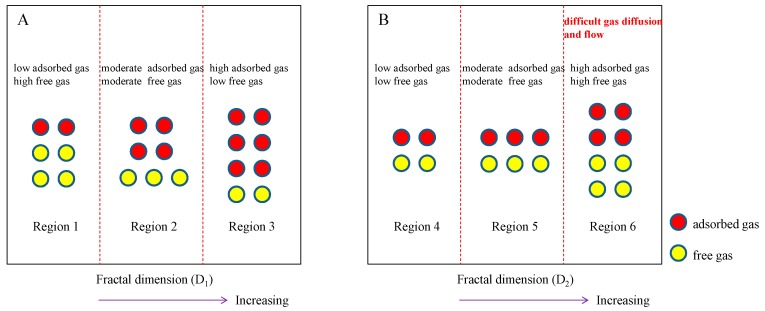
Relationships between shale gas storage capacity and fractal dimensions: (**A**) D_1_, (**B**) D_2._

­Therefore, the adsorbed gas storage capacity of shale would increase with the increase of D_1_ (Figure 15A). However, D_1_ shows a negative correlation with the pore volume which controls the free gas storage capacity of shale. As a consequence, the free gas storage capacity of shale decreases with the increase of D_1_ (Figure 15A). Note that the degree of positive correlation between D_1_ and the specific surface area is higher than the degree of negative correlation between D_1_ and the pore volume, thus the shale gas storage capacity (adsorbed gas storage capacity plus free gas storage capacity) are shown in the order: Region 1 < Region 2 < Region 3 (Figure 15A). It is indicated here that Region 1 refers to a region with relatively small D_1_ value (for example, 2.1–2.3), Region 2 refers to a region with relatively moderate D_1_ value (for example, 2.3–2.5), and Region 3 refers to a region with relatively large D_1_ value (for example, 2.5–2.7). 

In addition, the fractal dimension D_2_ is positively correlated with the specific surface area and pore volume, thus both the absorbed gas and free gas storage capacity of shale would increase when D_2_ increases (Figure 15B). Therefore, the shale gas storage capacity are shown in the order: Region 4 < Region 5 < Region 6 (Figure 15B). It is indicated here that Region 4 refers to a region with relatively small D_2_ value (for example, 2.65–2.70), Region 5 refers to a region with relatively moderate D_2_ value (for example, 2.70–2.75), and Region 6 refers to a region with relatively large D_2_ value (for example, 2.75–2.80). However, due to the strong negative relationship between D_2_ and the average pore diameter, the average pore diameter of shale would decreases with the increase of D_2_. It will enhance the complexity of pore structure, further hindering the natural gas diffusion and flow in shale (Region 6). In summary, it is suggested that shale with a large D_1_ (Region 3) and a moderate D_2_ (Region 5) has a strong capacity to store both adsorbed gas and free gas, and it also facilitates the exploitation and production of shale gas.

## 5. Conclusions 

In this paper, the pore structure and fractal characteristics of the Lower Jurassic lacustrine shale in the Sichuan Basin, SW China were investigated using a series of experiments and FHH theory. The following are the main conclusions from our study: 

(1) Pore types in shale are mainly OM and interP pores, along with a small amount of intraP pores, and that not all grains of OM have the same porosity. The BET surface areas of shale samples range from 4.10 to 8.38 m^2^/g with an average of 5.87 m^2^/g, DFT pore volumes range from 0.0076 to 0.0128 cm^3^/g with an average of 0.0098 cm^3^/g, and average pore diameters range from 5.56 to 10.48 nm with an average of 7.39 nm. 

(2) N_2_ adsorption experimental results show that shale has different adsorption characteristics at the relative pressure of 0–0.5 and 0.5–1. On this basis, two fractal dimensions D_1_ and D_2_ are obtained using FHH equation, with D_1_ values ranging from 2.2209 to 2.5648, and D_2_ values ranging from 2.6861 to 2.7964.

(3) The BET surface area shows a positive correlation with clay minerals content and quartz content, but no obvious relationship with TOC content. The DFT pore volume shows a positive correlation with TOC content and clay minerals content, but a negative relationship with quartz content. In addition, the average pore diameter shows a positive correlation with TOC content and a negative relationship with quartz content, but no obvious relationship with clay minerals content.

(4) Fractal dimension D_1_ is mainly closely associated with the specific surface area of shale, suggesting that D_1_ may represent the pore surface fractal dimension. Whereas fractal dimension D_2_ is sensitive to multiple parameters including the specific surface area, pore volume, and average pore diameter, suggesting that D_2_ may represent the pore structure fractal dimension.

(5) Shale with a large fractal dimension D_1_ and a moderate fractal dimension D_2_ has a strong capacity to store both adsorbed gas and free gas, and it also facilitates the exploitation and production of shale gas.

Other relevant experiments such as low-pressure CO_2_ adsorption, high-pressure mercury intrusion porosimetry (MIP), nuclear magnetic resonance (NMR) and nano-computed tomography (nano-CT) need to be further conducted to provide a more comprehensive understanding on pore structure and fractal characteristics of lacustrine shale.

## Figures and Tables

**Table 1:** Total organic carbon (TOC), mineralogical composition, and pore structure parameters of shale samples.

Sample ID	Depth (m)	TOC (%)	Mineralogical Composition Relative Percentage (%)					BET Surface Area (m^2^/g)	DFT Pore Volume (cm^3^/g)	Average Pore Diameter (nm)
			Quartz	Feldspar	Carbonates	Pyrite	Total Clays			
Y-1	3963.0	0.73	49.2	2.1	10.2	0	38.5	4.30	0.0076	7.80
Y-2	3964.2	0.39	37.1	1.9	21.3	0	39.7	5.31	0.0087	6.99
Y-3	3965.3	0.74	34.4	6.1	3.9	1.2	54.4	8.38	0.0109	5.56
Y-4	3966.1	0.54	49.1	2.7	4.2	0	44.0	6.67	0.0093	5.97
Y-5	3967.2	0.99	57.1	1.9	8.7	0	32.3	5.27	0.0081	6.80
Y-6	3968.4	0.97	36.4	5.2	10.1	1.2	47.1	5.76	0.0113	8.51
Y-7	3969.3	1.72	25.5	3.6	23.9	4.0	43.0	4.10	0.0101	10.48
Y-8	3970.1	1.63	41.0	6.2	10.9	0.6	41.3	6.26	0.0128	8.74
Y-9	3970.8	0.74	38.6	5.8	6.9	1.1	47.6	6.79	0.0091	5.70

**Table 2:** Fractal dimensions derived from the Frenkel–Halsey–Hill (FHH) model.

Sample ID	*P*/*P*_0_: 0–0.5			*P*/*P*_0_: 0.5–1		
	K_1_	D_1_ = 3 + K_1_	R^2^	K_2_	D_2_ = 3 + K_2_	R^2^
Y-1	−0.4796	2.5204	0.8953	−0.2878	2.7122	0.9947
Y-2	−0.4576	2.5424	0.9250	−0.2635	2.7365	0.9842
Y-3	−0.5045	2.4955	0.8797	−0.2036	2.7964	0.9807
Y-4	−0.4940	2.5060	0.8807	−0.2110	2.7890	0.9689
Y-5	−0.4352	2.5648	0.9378	−0.2433	2.7567	0.9839
Y-6	−0.6567	2.3433	0.9174	−0.2705	2.7295	0.9676
Y-7	−0.7791	2.2209	0.9173	−0.3139	2.6861	0.9541
Y-8	−0.6805	2.3195	0.9526	−0.2760	2.7240	0.9623
Y-9	−0.5368	2.4632	0.8238	−0.2141	2.7859	0.9670
